# Endogenous Collagenases Regulate Osteoclast Fusion

**DOI:** 10.3390/biom10050705

**Published:** 2020-05-01

**Authors:** Hyo Jeong Kim, Youngkyun Lee

**Affiliations:** 1 Department of Biochemistry, School of Dentistry, Kyungpook National University, Daegu 41940, Korea; hjsm48@nate.com; 2 Institute for Hard Tissue and Bio-tooth Regeneration (IHBR), School of Dentistry, Kyungpook National University, Daegu 41940, Korea

**Keywords:** collagenase, osteoclast, fusion, ERK

## Abstract

The precise regulation of osteoclast differentiation and function is crucial for the maintenance of healthy bone. Despite several reports of collagenase expression in bone tissues, the precise isoform expression as well as the role in osteoclasts are still unclear. In the present report, the expression of matrix metalloprotease (MMP)8 and MMP13 was confirmed in mouse bone marrow macrophage osteoclast precursors. The mRNA and protein expressions of both collagenases were significantly reduced by receptor activator of nuclear factor κB ligand (RANKL) stimulation. Notably, either inhibition of MMP expression by siRNA or treatment of cells with collagenase inhibitor Ro 32-3555 significantly augmented osteoclast fusion and resorption activity without affecting the osteoclast number. The inhibition of collagenase by Ro 32-3555 increased the expression of osteoclast fusion genes, *Atp6v0d2* and *Dcstamp*, without affecting nuclear factor of activated T-cells, cytoplasmic 1 (NFATc1) protein expression. The enhanced osteoclast fusion by collagenase inhibition appears to be mediated through an extracellular signal regulated kinase (ERK)-dependent pathway. Collectively, these data provide novel information on the regulation of osteoclast fusion process.

## 1. Introduction

Osteoclasts are sole bone-resorbing cells found in the skeletons of higher vertebrates originated from the macrophage/monocyte lineage of hematopoietic stem cells [1]. The concerted actions of bone-forming osteoblasts and osteoclasts are pivotal for the maintenance of healthy bones through a process called “bone remodeling”, in which old or damaged bones are replaced by new bones [2]. The unbalance in the bone remodeling, in many cases uncontrolled osteoclast differentiation and activation, often leads to pathological skeletal conditions seen in osteoporosis, rheumatoid arthritis, and periodontitis [3,4,5]. Thus, a better understanding of osteoclast biology is crucial for the development of intervention strategies targeting osteoclasts for such diseases. The macrophage-colony stimulating factor (M-CSF) furnished by bone microenvironment supports the survival of osteoclast precursors, while receptor activator of nuclear factor κB ligand (RANKL) induces their differentiation. Osteoclast differentiation is a multi-step process. In early phase of osteoclastogenesis, the precursor cells are committed by multiple RANKL-dependent signaling events including mitogen-activated protein kinases (MAPKs), nuclear factor κB (NFκB), and nuclear factor of activated T-cells, cytoplasmic 1 (NFATc1), the key transcription factor for osteoclastogenesis [6,7,8]. The committed mononuclear osteoclasts fuse to form mature, fully functional multinuclear osteoclasts. It has been suggested that the roles of vacuolar ATPase (v-ATPase) and dendritic cell-specific transmembrane protein (DC-STAMP) are essential for cell–cell fusion [9]. However, the information on the regulation of this later phase of osteoclastogenesis is considerably lacking, compared with the vast amount of knowledge regarding early signaling events [9,10].

Matrix metalloproteases (MMPs) are family of proteolytic enzymes encoded by 24 genes in human [11] that are involved in diverse cellular and pathological events involving matrix remodeling [12]. Among those MMPs, collagenases are comprised of MMP1, 8, and 13 according to classification based on structural similarity and substrate specificity. The expression of collagenases has been observed in various cells in skeletal system, including osteoblasts and osteoclasts [13,14,15,16]. Notably, the role of collagenases in bone development and remodeling was demonstrated in MMP13-deficient mice, in which the endochondral ossification as well as the appearance of osteoclasts in the primary ossification centers are significantly delayed during embryogenesis [17,18]. In a more recent study, the role of MMP13 in adult skeleton was attributed to osteocytic perilacunar remodeling required for the maintenance of bone quality [19]. MMP8-deficiency led to a more severe alveolar bone loss in *Porphyromonas gingivalis* infection model [20] and increased joint inflammation and bone loss in serum transfer arthritis model [21]. However, the expression and function of collagenases, i.e., MMP1, 8, and 13, in osteoclasts are yet to be thoroughly studied. 

In the present study, the role of collagenases during osteoclast differentiation was examined in mouse bone marrow macrophages as osteoclast precursors in vitro as well as in calvarial bones. The data indicated that MMP8 and 13 might be involved in endogenous inhibition of osteoclast fusion, suggesting novel roles of collagenases in osteoclasts. 

## 2. Materials and Methods

### 2.1. Animals and Cells

Osteoclast precursors were isolated from the femora and tibiae of female ICR mice (5-week-old) obtained from Central Laboratory Animal Inc. (Seoul, Korea). Bone marrows were flushed with Eagle’s minimal essential medium, α-modification (α-MEM) (Welgene, Daegu, Korea) using 1 mL syringes. After removing the red blood cells, the collected bone marrow cells were incubated overnight in the presence of 10% fetal bovine serum (FBS) (Life Technologies, Carlsbad, CA, USA) and adherent cells on plastic were discarded. Bone marrow macrophages (BMMs) were obtained by culturing the cells further for 3 days with 20 ng/mL M-CSF (PeproTech, Rocky Hill, NJ, USA). All animal experimental protocols were approved by the committees on the care and use of animals in research at Kyungpook National University. 

### 2.2. Reagents and Antibodies

Recombinant human soluble RANKL and M-CSF were purchased from PeproTech. Ro 32-3555 was obtained from Tocris Bioscience (Bristol, UK). Lipopolysaccharide (LPS) from *E. coli* O111:B4 was purchased from Sigma-Aldrich (St. Louis, MO, USA). Antibody against NFATc1 (7A6) was from BD Pharmingen (Franklin Lakes, NJ, USA). Antibody against Rho A (119) and siRNA duplexes targeting MMP8 (sc-35950) and MMP13 (sc-41560) were from Santa Cruz Biotechnology (Santa Cruz, CA, USA). Anti-NFκB antibody (D14E12) was obtained from Cell Signaling Technology (Beverly, MA, USA). Anti β-actin (AC-74) was from Sigma-Aldrich. The siRNAs against ERK1 (s77117) and ERK2 (s77104) were purchased from Thermo Fisher Scientific (Waltham, MA, USA). Chemical inhibitors including PD 98059, caffeic acid phenethyl ester, and cyclosporine A were purchased from Merck (Darmstadt, Germany). All other chemicals were obtained from Sigma unless otherwise specified.

### 2.3. Osteoclast Differentiation and Measurement of Cell Size

BMMs, used as osteoclast precursors, were seeded on 48-well plates (2 × 10^4^ cells/well) and incubated with 20 ng/mL M-CSF and 100 ng/mL RANKL with media change at every 2 days. At 4 days after culture, cells were fixed and subjected to tartrate-resistant acid phosphatase (TRAP) staining using leukocyte acid phosphatase kit (Sigma). Osteoclasts were observed under Olympus BX53 light microscope with a 10×/0.30 UplanFL N objective lens (Olympus, Center Valley, PA, USA) equipped with a DP73 digital camera system, with image capture using CellSens software (Olympus). The average size of osteoclasts was determined by measuring 20 largest cells in the field using OsteoMeasure software (OsteoMetrics, Decatur, CA, USA).

### 2.4. Co-Culture of BMMs with Calvarial Cells

Mouse calvarial cells were isolated from neonatal ICR mice (1 day old) by treatment of mouse calvariae with dispase and collagenase [22]. After overnight culture of calvarial cells (10^4^/well in 48-well plates), BMMs were added (2 × 10^4^ cells/well) and were further cultured for 7 days with 10 nM vitamin D_3_ and 1 μM prostaglandin E_2_ (PGE_2_) before staining for TRAP activities.

### 2.5. In Vitro Osteoclast Resorption Assay

BMMs were cultured on dentin discs (Immunodiagnostic Systems, Boldon, UK) with M-CSF and RANKL for 7 days. After cell removal by incubation in 0.5% Triton X-100 and brief sonication, resorption pits were stained by applying hematoxylin solution with cotton swabs. 

### 2.6. The siRNA Knock-Down

BMMs seeded on 48-well plates (2 × 10^4^ cells/well) were transfected with siRNAs targeting either control, MMP8, or MMP13, ERK1, and ERK2 using the HiPerFect transfection reagent (Qiagen, Hilden, Germany). Cells were further cultured for 48 h in the presence of 20 ng/mL M-CSF before the measurement of MMP or ERK expression.

### 2.7. Quantitative Real-Time PCR

BMMs were lysed in TRIzol reagent (Thermo Fisher Scientific) to extract RNA. After reverse transcription using 1 μg of RNA, cDNAs were amplified with SYBR green master mix (Thermo Fisher Scientific) in optical tubes using a 7500 real-time PCR system (Applied Biosystems, Foster City, CA, USA). The mRNA expression relative to that of *Hprt1* was calculated by the 2^–ΔΔCt^ method [23].

### 2.8. Measurement of Osteoclasts in Calvarial Bones

Collagen sheets (Zimmer Biomet, Palm Beach Gardens, FL, USA) were cut into pieces (10 × 10 mm, 1mm thick) and soaked in phosphate buffered saline (PBS), LPS (100 μg), or Ro 32-3555 (1 nmol). These collagen slabs were inserted onto the calvariae of 5-week-old female C57BL/6 mice. At 5 days after operation, calvariae were removed, fixed, and decalcified before processing for paraffin embedding. Tissue sections of 5 μm thickness were prepared using a Leica RM 2245 microtome (Leica Microsystems, Bannockburn, IL) and subsequently subjected to TRAP staining. Histomorphometric analyses for the number of osteoclasts per bone perimeter (N. Oc/B. Pm.), osteoclast perimeter per osteoclast (OC Pm/N. OC), and eroded surface per bone surface (ES/BS) were performed using OsteoMeasure software (OsteoMetrics, Decatur, CA, USA). 

### 2.9. Statistics

All data presented are representative of at least three experiments performed in triplicates unless otherwise specified. A two-tailed Student’s t-test (comparison between 2 samples) or one-way ANOVA followed by Student Knewman-Keuls *post hoc* test (comparison among multiple samples) was used to determine the differences between results in which *p* < 0.05 were considered statistically significant. The Mann–Whitney *U* test (2 groups) and Kruskal–Wallis rank sum test with Dunn’s *post hoc* analysis (more than 2 groups) were used for the comparison of osteoclast size.

## 3. Results

### 3.1. The Role of Endogenous Collagenases in Osteoclastogenesis

**Figure 1:**
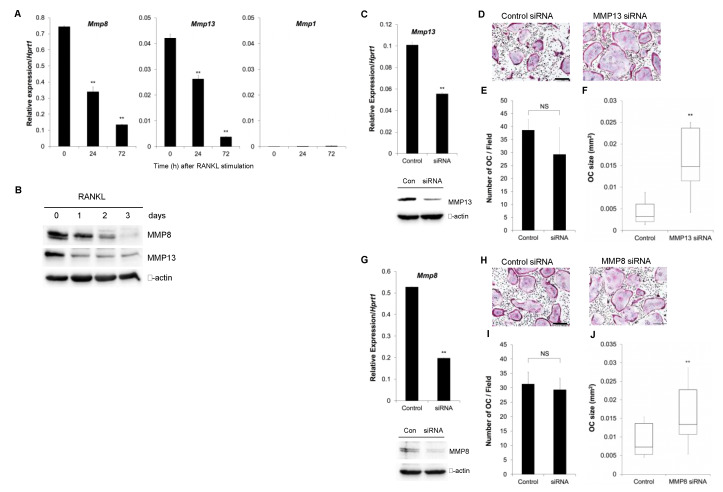
The role of matrix metalloprotease (MMP)8 and MMP13 on osteoclast differentiation. (**A**) Bone marrow macrophages (BMMs) were cultured in the presence of macrophage-colony stimulating factor (M-CSF) and receptor activator of nuclear factor κB ligand (RANKL) for 3 days, and the expression of collagenases was determined by real-time RT-PCR analysis. (**B**) BMMs were cultured as in (**A**) were lysed and subjected to Western blot analyses. (**C**) BMMs were cultured in the presence of MMP13-siRNA for 2 days and the expression of MMP13 was confirmed by real-time RT-PCR analysis (upper panel) and Western blot (lower panel). (**D**) Cells in (**C**) were further cultured in the presence of M-CSF and RANKL for 5 days and stained for tartrate-resistant acid phosphatase (TRAP) activities. (**E**) The number of TRAP-positive osteoclasts with more than 3 nuclei was counted from experiments in (**D**). (**F**) The size of osteoclasts was measured from the experiments in (**D**). (**G**) The MMP8 mRNA and protein expression were examined after culturing BMMs with MMP8-siRNA for 2 days. (**H**) Cells in (**G**) were further cultured in the presence of M-CSF and RANKL for 5 days and stained for TRAP activities. (**I**) The number of osteoclasts was counted from experiments in (**H**). (**J**) The osteoclast size from the experiments in (**H**) was measured. Data are mean ± SD of triplicate experiments representative of three independent experiments. **, *p* < 0.01 versus control. Scale bars indicate 100 μm.

In an effort to delineate the roles of endogenous collagenases during osteoclastogenesis, the expression of MMP1, MMP8, and MMP13 was examined in BMMs treated with M-CSF and RANKL (Figure 1A). The expression levels of both MMP8 and MMP13 were highest at day 0 and significantly decreased during osteoclast differentiation. The expression of MMP1 was almost negligible in mouse BMMs. To further examine the expression of collagenases in protein level, Western blot analyses were performed. As shown in Figure 1B, the expression of both MMP8 and MMP13 significantly decreased over time after the addition of RANKL in osteoclast precursors. To evaluate the direct effect of collagenases on osteoclast differentiation, MMP13 was knocked down. Figure 1C shows that MMP13 expression was efficiently suppressed both in mRNA (upper panel) as well as in protein level (lower panel) by the treatment of BMMs with a siRNA targeting MMP13. When these cells were incubated in the presence of M-CSF and RANKL, there was an apparent increase in the size of TRAP-positive multinuclear osteoclasts after MMP13 knock-down (Figure 1D). Interestingly, however, there was no significant change in the number of osteoclasts (Figure 1E). The box and whisker plot for the distribution of osteoclast size in Figure 1F indicates that the median size of osteoclasts is more than four times larger in MMP13 knock-down cells compared with control. Similarly, MMP8 was also effectively knocked down in mouse BMMs both in mRNA and protein level using a siRNA targeting MMP8 (Figure 1G). BMMs treated with control or MMP8 siRNA were stained for TRAP activity after culturing with M-CSF and RANKL (Figure 1H). Although the number of osteoclasts formed exhibited no significant difference (Figure 1I), the median size of the mature osteoclasts was almost twice larger in MMP8 knock-down cells compared with control (Figure 1J).

### 3.2. The Effect of Collagenase Inhibition on Osteoclast Size

**Figure 2:**
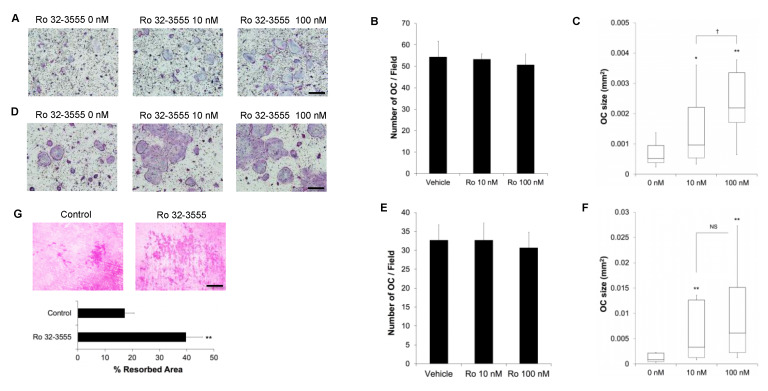
The effect of collagenase inhibition on osteoclast size. (**A**) BMMs were cultured with M-CSF and RANKL for 5 days in the presence of a collagenase inhibitor Ro 32-3555. Osteoclasts were stained for TRAP activities. (**B**) The number of osteoclasts was counted from the experiments in (**A**). (**C**) The size of osteoclasts was measured form the experiments in (**A**). (**D**) BMMs were co-cultured with calvarial cells for 7 days in the presence of vitamin D_3_ and PGE_2_ to induce osteoclast differentiation. Cells were stained for TRAP activities. (**E**) The number of osteoclasts was counted from the experiments in (**D**). (**F**) The osteoclast size was measured from the experiments in (**D**). (**G**) BMMs were cultured on dentin discs for 7 days with M-CSF and RANKL in the presence or absence of 100 nM Ro 32-3555. The resorption pits were stained with hematoxylin. Data are mean ± SD of triplicate experiments representative of three independent experiments. *, *p* < 0.05. **, *p* < 0.01 against control. †, *p* < 0.05 between two concentrations. Scale bars indicate 100 μm.

To further delineate the role of collagenases during osteoclastogenesis, BMMs were treated with a collagenase inhibitor Ro 32-3555 and cultured with M-CSF and RANKL. As shown in Figure 2A, TRAP staining of these cells revealed that the treatment with 10 nM inhibitor was enough to induce the generation of significantly larger osteoclasts. In consistence with the results from collagenase knock-down cells, the addition of Ro 32-3555 did not affect the number of osteoclasts generated (Figure 2B). On the other hand, the addition of 10 nM Ro 32-3555 resulted in the formation of osteoclasts almost twice larger than vehicle-treated cells (Figure 2C). Increasing the concentration of Ro compound to 100 nM triggered the generation of even larger osteoclasts (almost four-times compared with control). The effect of collagenase inhibition on osteoclast size was further tested using the calvarial cell-osteoclast co-culture system (Figure 2D). Similar to the results obtained with BMMs, the addition of Ro compound to the co-culture of these cells also induced the formation of significantly larger osteoclasts without any effect on the number of osteoclasts generated (Figure 2E). The median size of osteoclasts was four-times and seven-times larger compared with vehicle-treated control when cells were treated with 10 nM and 100 nM Ro compound, respectively (Figure 2F). Finally, the bone resorption activities of osteoclasts were examined by culturing BMMs on dentin discs with M-CSF and RANKL in the presence or absence of 100 nM Ro 32-3555. As shown in Figure 2G, the resorbed area was significantly larger (more than twice) compared with control when cells were treated with Ro compound.

**Figure 3:**
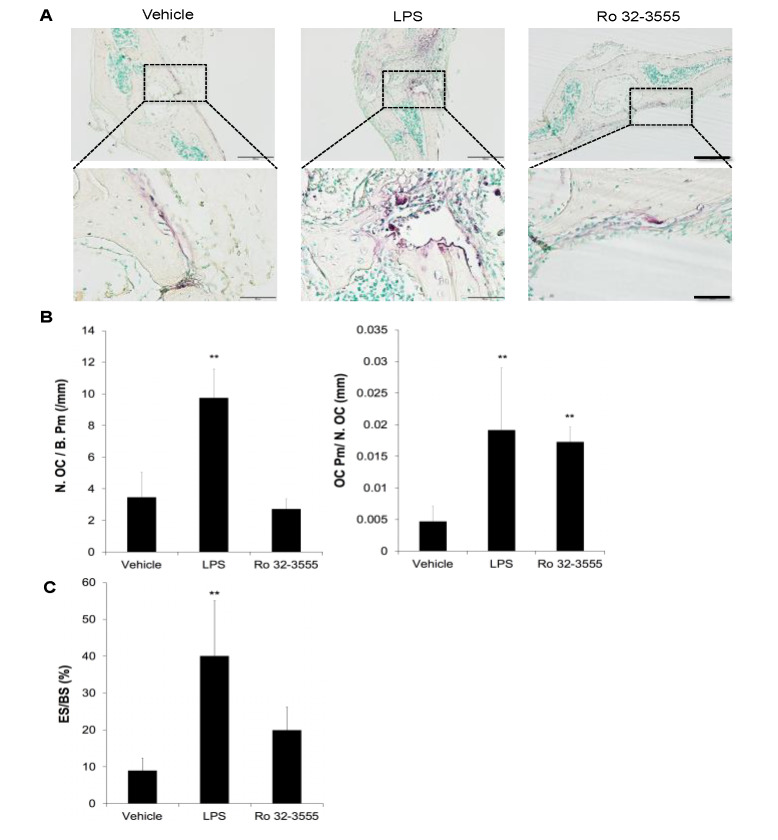
The effect of collagenase inhibition on osteoclastogenesis in calvarial bones. (**A**) Collagen sponges containing PBS, lipopolysaccharide (LPS), or Ro 32-3555 were surgically inserted onto mouse calvariae. At 5 days after stimulation, mice were sacrificed, and the calvarial bone sections were prepared and stained for TRAP-activities. Bone area on which collagen sponges were inserted was photographed. Scale bars indicate 200 mm and 50 mm in upper and lower panels, respectively. (**B**) Histomorphometry analyses were performed to examine osteoclast number (N. OC/B. Pm) and osteoclast size (OC Pm/N. OC). (**C**) Both osteoclast-positive and -negative resorption lacunae were measured to calculate eroded surface per bone surface (ES/BS). Data are mean ± SD obtained from 5 samples. **, *p* < 0.01 against vehicle-treated control.

Since the inhibition of collagenase activities dramatically increased osteoclast size in vitro, the effect of Ro 32-3555 on bone tissues was further tested. Collagen sponges soaked in PBS, Ro compound, or LPS were inserted onto mouse calvariae to induce osteoclastogenesis in vivo. LPS served as a positive control since it is known to potently stimulate osteoclastogenesis in vitro and induce significant bone resorption in mouse calvariae [24,25]. Tissue sections were stained for TRAP activity and analyzed for osteoclast number and size. As shown in Figure 3A, administration of LPS dramatically increased the number of TRAP stained cells with concomitant appearance of significantly larger osteoclasts compared with control. Notably, the addition of Ro 32-3555 seemed to exert no effect on the number of TRAP-positive osteoclasts. However, the existence of conspicuously larger osteoclasts was observed in bone tissues after treatment with Ro compound. Histomorphometry analyses further confirmed significantly increased osteoclast number in LPS-treated calvariae, while no such effect was detected when tissues were treated with Ro 32-3555 (Figure 3B, left panel). However, both LPS- and Ro compound-treated tissues exhibited significantly higher osteoclast perimeter per osteoclast (OC pm/N. OC), suggesting distinctively larger osteoclasts in these specimens (Figure 3B, right panel). To estimate the osteoclast activities in these samples, eroded surface per bone surface (ES/BS) was measured (Figure 3C). While LPS dramatically increased the eroded surface, Ro 32-3555 did not enhance bone resorption to a statistically significant level, although the mean ES/BS was more than twice larger compared with control samples.

### 3.3. The Signaling Pathways by which the Inhibition of Collagenase Activities Regulate Osteoclast Size

**Figure 4:**
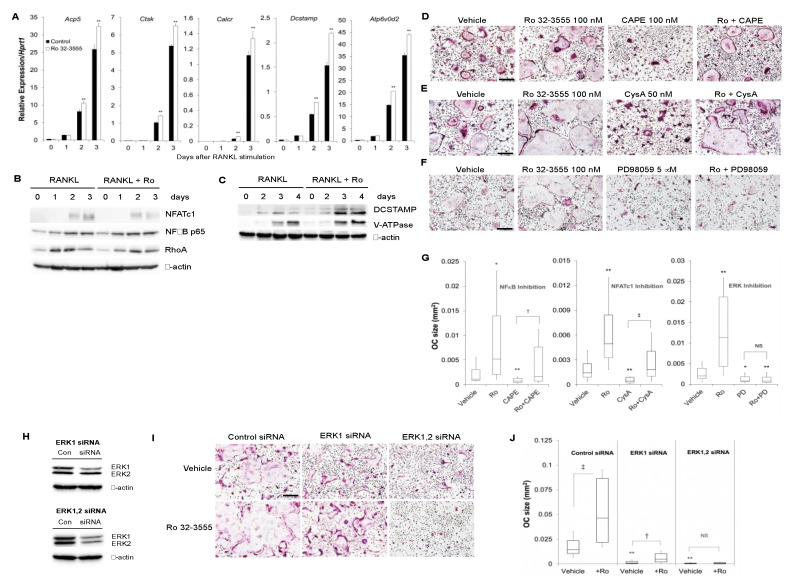
Signaling pathways involved in the increased osteoclast size after collagenase inhibition. (**A**) Control or Ro 32-3555-treated (100 nM) cells were subjected to the real-time RT-PCR analyses for the expression of osteoclast marker genes, *TRAP (Acp5)*, *Cathepsin K (Ctsk)*, *Calcitonin Receptor (Calcr)*, *Dcstamp*, *and v-ATPase (Atp6v0d2)*. (**B** and **C**) BMMs were cultured with M-CSF and RANKL for indicated days in the absence or presence of 100 nM Ro 32-3555. Western blot analyses were performed using cell lysates to examine the expression of proteins involved in osteoclast differentiation and fusion. (**D**–**F**) BMMs were cultured for 5 days with M-CSF and RANKL. The effect of NFκB inhibitor caffeic acid phenetyl ester (CAPE) (**D**), nuclear factor of activated T-cells, cytoplasmic 1 (NFATc1) inhibitor cyclosporin A (**E**), and ERK inhibitor PD98059 (**F**) on osteoclast size in Ro 32-3555-stimulated cells was tested by TRAP staining. (**G**) The osteoclast size was measured from experiments in (**D**–**F**). (**H**) BMMs were transfected with ERK1 siRNA (upper panel) or ERK1 and ERK2 siRNA (lower panel). The expression of ERK1 and ERK2 was examined by Western blot after 48 h incubation in the presence of M-CSF. (**I**) Cells in (**H**) were incubated with M-CSF and RANKL for 4 days in the presence or absence of 100 nM Ro 32-3555 before TRAP staining. (**J**) The size of osteoclasts in (**I**) was measured. Data are mean ± SD representative of three independent experiments. *, *p* < 0.05. **, *p* < 0.01 versus control. †, *p* < 0.05. ††, *p* < 0.01 between inhibitor-treated groups. Scale bars indicate 100 μm.

To dissect the molecular mechanisms involved in the regulation of osteoclast size by collagenase inhibition, the expression of osteoclast marker genes was first examined after the treatment of cells with Ro 32-3555 (Figure 4A). The addition of Ro compound induced a slight increase in the expression of osteoclast marker genes such as *Acp5* (TRAP), *Ctsk* (cathepsin K), and *Calcr* (calcitonin receptor) compared with vehicle-treated control. In addition, Ro 32-3555 also elicited a similar slight increase in the expression of osteoclast fusion markers, *Dcstamp* and *Atp6v0d2* (v-ATPase). The expression levels of osteoclast marker proteins were further tested by Western blot analyses (Figure 4B). The expression of NFATc1 was not remarkably affected by the treatment of cells with Ro 32-3555. Similarly, the expression of NFκB p65 as well as ras homologue gene family member A (RhoA) did not notably change in Ro compound-treated cells compared with control. Figure 4C confirms the increased expression of both DC-STAMP and v-ATPase proteins in Ro 32-3555-treated cells, in accordance with their increased mRNA expression levels. To further explore the signaling pathways involved in the increased osteoclast size following collagenase inhibition, the effect of pharmacological inhibitors was examined. The concentration of inhibitors was chosen not to completely shut down osteoclast differentiation to evaluate the contribution of each signal to the regulation of osteoclast size. In Figure 4D, the treatment of Ro compound induced significantly larger osteoclast size compared with control, while an NFκB inhibitor caffeic acid phenetyl ester (CAPE) [26] alone dramatically reduced it. However, Ro compound still greatly induced the formation of significantly larger osteoclasts even in the presence of CAPE (Figure 4G, left panel). Similarly, the inhibition of NFATc1 by cyclosporin A (CysA) [27] triggered the generation of small osteoclasts, while the addition of RO 32-3555 completely reversed the effect of CysA (Figure 4E,G, middle panel). Notably, however, Ro compound did not exert any effect on osteoclast size when cells were treated with an ERK pathway inhibitor, PD98059 [28] (Figure 4F,G, right panel). To further corroborate the role of ERK signaling in Ro compound-mediated osteoclast fusion, ERK expression was knocked-down in osteoclast precursors using siRNAs specific for ERK1 or ERK2 (Figure 4H). Western blot experiments confirmed the reduced expression of ERK1 (upper panel) and both ERK1 and ERK2 (lower panel) in BMMs treated with ERK1 siRNA and ERK1 plus ERK2 siRNA, respectively. When these cells were treated with Ro-32-3555 in the presence of M-CSF and RANKL, significant increase in the osteoclast size was observed in the control siRNA- and ERK1 siRNA-treated cells (Figure 4I,J). However, no such enhanced fusion was detected when both ERK1 and ERK2 expressions were knocked down.

## 4. Discussion

The collagenase activities in bone have been known for a long time [29], among which MMP13 is most well-recognized to be expressed in hypertrophic chondrocytes and osteoblasts [13,30,31]. Indeed, mice deficient in MMP13 showed abnormal growth plate development and endochondral bone formation [17,18]. Although less well-studied than MMP13, the existence of MMP8 was also suggested in chondrocytes, osteoblasts, and osteocytes [14]. However, the expression of collagenases in osteoclasts is controversial depending on research groups and experimental tools used [15]. Furthermore, the roles of collagenases in osteoclast differentiation and function still remains to be elucidated. In the present report, we demonstrated that the inhibition of endogenous collagenase expression or activities dramatically increased osteoclast fusion without affecting osteoclast differentiation. These results were reproduced in in vitro culture of mouse BMMs as well as in co-culture of calvarial cells and BMMs. Furthermore, similar increase of osteoclast size, without change in osteoclast number, was observed in mouse calvariae when collagenase inhibitor was administrated in vivo. Since the expression of both MMP8 and MMP13 (predominant collagenases in mouse BMMs, (Figure 1) considerably decreased by RANKL treatment, it is believed that the reduction of collagenases is a prerequisite for efficient osteoclast fusion. For the MMP8 or MMP13 expression in chondrocytes, transcriptional regulation by NFkB, activator protein 1 (AP-1), and Runx2 was suggested as possible mechanisms [32,33]. The signaling pathways involved in the RANKL-dependent regulation of MMP expression in osteoclast precursors need to be elucidated by further studies.

The fusion of TRAP-positive mononuclear osteoclast precursors is essential to form fully functional mature osteoclasts. Although the precise mechanisms are yet to be completely unraveled, several factors are known to regulate the fusion process. Among those, the roles of DC-STAMP and v-ATPase are most well-known. The expression of these proteins dramatically increases at the time of osteoclast fusion, rendering these molecules considered as RANKL-dependent fusogens [9]. Indeed, *Atp6v0d2*-deficient mice exhibited significantly defective osteoclast fusion without apparent reduction in osteoclast commitment [34]. Similarly, mice deficient in *Dcstamp* developed osteopetrosis due to defective osteoclast fusion [35]. In the current experiments, the treatment of osteoclast precursors with a collagenase inhibitor Ro 32-3555 enhanced the expression of these fusogenic genes (Figure 4). Thus, it is believed that the inhibition of collagenase might accelerate osteoclast fusion through the modulation of v-ATPase and DC-STAMP expression, at least in part. To further delineate the signaling mechanism underlying enhanced osteoclast fusion by collagenase inhibition, the expression of principal osteoclast transcription factors, NFATc1 and NFκB, was scrutinized in osteoclast precursors in the presence of Ro 32-3555 (Figure 4). Notably, both *Atp6v0d2* and *Dcstamp* are known as downstream genes of *Nfatc1* [36], the master transcription factor for osteoclastogenesis. Interestingly, the collagenase inhibitor did not significantly increase NFATc1 protein expression, suggesting that the enhanced fusion by Ro 32-3555 might be mediated by an NFATc1-independent process. Further supporting this notion, Ro 32-3555 still significantly augmented osteoclast fusion in the presence of an NFATc1 inhibitor, cyclosporin A (Figure 4). Similarly, the size of osteoclast was conspicuously larger when BMMs were treated with Ro 32-3555 in the presence of NFκB inhibitor, implicating that NFκB is also not involved in the augmented fusion process by collagenase inhibition. On the other hand, the presence of ERK inhibitor PD 98,059 completely prevented the fusogenic effect of Ro 32-3555. These results are congruent with the suggested role of ERK in osteoclast fusion. He et al. reported that the deficiency of *Erk1* prompted significantly smaller osteoclasts from bone marrow mononuclear cells in vitro [37]. Conversely, the adenoviral infection of pre-osteoclasts with a constitutive active form of mitogen-activated protein kinase (MEK) dramatically enhanced osteoclast fusion [38]. Interestingly, current experiments using siRNAs suggested that the increased fusion by Ro 32-3555 is more likely dependent on ERK2 rather than ERK1 (Figure 4). Although the dramatic decrease of osteoclast fusion in the presence of ERK1 siRNA could be recovered by the collagenase inhibitor, additional ERK2 siRNA completely abrogated the Ro compound-dependent increase of osteoclast size. Previous reports indicated that the inhibition of ERK pathway by the treatment of osteoclast precursors with a MEK inhibitor (U0126 or PD98059) significantly suppressed the expression of *Dcstamp* [39,40]. Thus, potential mechanisms behind the ERK pathway-dependent enhancement of osteoclast fusion by Ro 32-3555 in current study might involve *Dcstamp*, considering the suggested pivotal role during the fusion of osteoclasts [9,35,40].

In spite of the direct enhancement of osteoclast fusion by the inhibition of collagenase expression or activity shown in the present study, the exact role as well as underlying mechanisms in the context of bone microenvironment still need to be more extensively studied. The enhanced resorption activity of osteoclasts with concomitantly increased size by collagenase inhibition (Figure 2) is in good accordance with previous reports that larger osteoclasts with more nuclei tend to have higher resorption activity compared with smaller ones [41,42,43]. These observations raise the possibility that the reduced expression or activities of osteoclast collagenases may contribute to the increased bone remodeling or exacerbated pathological bone loss. However, there exists a contradicting report on the role of MMP13 in osteoclast fusion. Recently, Fu et al. studied the role of MMP13 on osteoclastogenesis in a tumor–bone microenvironment using multiple myeloma cells [44]. Notably, multiple myeloma-derived MMP13 promoted the fusion of osteoclasts originated from mouse bone marrow cells in the presence of M-CSF and RANKL, proposing a role of exogenous collagenase. Interestingly, the authors suggested that the fusogenic action was independent of its protease activity. Thus, further studies considering the localization and origin of collagenases in the context of bone microenvironment are required to fully understand the role of collagenases in osteoclast fusion process.

## 5. Conclusions

We discovered that the down-regulation of endogenous expression and activities of collagenases in mouse osteoclast precursors stimulated osteoclast fusion in an ERK pathway-dependent manner with a concomitant increase in the v-ATPase and DC-STAMP expression. These data not only provide novel insights into the mechanics of osteoclast fusion but also suggest a very convenient and simple model to study the fusion process of osteoclast precursors.
